# 4303 Optimization of primary septal-hippocampal co-cultures to study central cholinergic synapse formation and dysfunction

**DOI:** 10.1017/cts.2020.86

**Published:** 2020-07-29

**Authors:** Sarra Djemil, Claire R. Ressel, Daniel T.S. Pak

**Affiliations:** 1 Georgetown - Howard Universities

## Abstract

OBJECTIVES/GOALS: Septal cholinergic innervation to the hippocampus is critical for normal learning and memory and is severely degenerated in Alzheimer’s disease. To understand the molecular events underlying this loss, we optimized a primary septal-hippocampal co-culture system that facilitates study of central cholinergic synapses. METHODS/STUDY POPULATION: We developed an optimized *in vitro* septal-hippocampal co-culture system modified from previous published protocols. Briefly, hippocampal and septal tissue were harvested from embryonic day 19 (E19) Sprague-Dawley rats, digested with 0.1% trypsin, and an equal number of cells from each region plated onto coverslips coated with poly-D-lysine and laminin at a final density of 300 cells/mm^2^. We use immunostaining with validated primary antibodies and a fluorescent binding assay, together with confocal microscopy, to determine the structure of cholinergic synapses that are 1) native, 2) mammalian, 3) CNS derived, 4) comprised of physiological synaptic partners, and 5) developmentally mature. RESULTS/ANTICIPATED RESULTS: After DIV21, co-cultures maintained a healthy morphology. A subpopulation of neurons strongly expressed the cholinergic markers vesicular ACh transporter (vAChT), choline acetyltransferase (ChAT), and the high-affinity choline transporter (ChT1), whereas most neurons lacked vAChT expression and were presumably glutamatergic or GABAergic. The percentage of cholinergic neurons in the co-culture attained up to ~5-7%, depending on conditions such as embryo age at dissection or ratio of septal to hippocampal cells. We also report on cholinergic synapse structure by examining postsynaptic markers (excitatory and inhibitory) and staining for nicotinic acetylcholine receptor subunits. DISCUSSION/SIGNIFICANCE OF IMPACT: Primary septal-hippocampal co-cultured neurons have not been exploited extensively in the field, perhaps due to the difficulty in maintaining such cultures for extended periods. Here, we optimized an *in vitro* septal-hippocampal co-culture system, a powerful tool to comprehensively analyze central cholinergic synapse formation and dysfunction.

